# Supplementary material for: Genome-wide signatures of local adaptation among seven stoneflies species along a nationwide latitudinal gradient in Japan
Source: BMC Genomics. 2019 Jan 24;20:84. doi: 10.1186/s12864-019-5453-3 (PMC6346529; doi:10.1186/s12864-019-5453-3)
Supplement: Supplementary file 1 — Table S1. Sampling sites and associated meteorological data. Table S2. Number of individuals per sampling site. Geographical Regions: M, Matsuyama; G, Gifu; S, Sendai; and Sa, Sapporo. Sampling site codes are shown as described in Additional file 1. Appendix S1. de novo assembly filtering and post-assembly filtering. Table S3. Number of loci by different parameter combinations evaluated for the de novo assembly. Table S4. Number of loci after final data filtering using the population program of the STACKS program. Table S5. Fst values after final data filtering using the population program of the STACKS program. Table S6. Numbers reads per species per region using the parameter combination m3 M2 n2 for the de novo assembly after the population program by STACKS. Table S7. Numbers of loci per species per region using the parameter combination m3 M2 n2 for de novo assembly after population program by STACKS. Figure S1. Flow chart of data filtering steps. (DOCX 4012 kb) [file 12864_2019_5453_MOESM1_ESM.docx]

**Supplementary information**

**Genome-wide signatures of local adaptation among seven stream stoneflies along a nationwide latitudinal gradient in Japan**

**Maribet Gamboa & Kozo Watanabe**

Department of Civil and Environmental Engineering, Ehime University, 790-0871 Matsuyama, Japan

gamboa@cee.ehime-u.ac.jp

**Additional file 1: Table S1.** Sampling sites and associated meteorological data

| Geographical region | Site | Code | Altitude (m) | Longitude | Latitude | Precipitation (mm) | Water level (m) | Discharge (m^3^/s) | Snow cover (cm) | Water temp. (°C) | Air temp. (°C) |
| --- | --- | --- | --- | --- | --- | --- | --- | --- | --- | --- | --- |
|  | Ishite River | M1 | 277 | 132.864 | 33.899 | 0.11 | 1.28 | 0.39 | 2 | 12.3 | 10.3 |
|  | Ishite River | M2 | 270 | 132.864 | 33.897 | 0.11 | 1.28 | 0.39 | 2 | 12 | 10.3 |
| Matsuyama | Ishite River | M3 | 269 | 132.864 | 33.897 | 0.11 | 1.28 | 0.39 | 2 | 12.1 | 10.3 |
|  | Ishite River | M4 | 246 | 132.847 | 33.896 | 0.10 | 1.20 | 0.39 | 2 | 12 | 10.3 |
|  | Ishite River | M5 | 156 | 132.847 | 33.876 | 0.11 | 1.20 | 0.39 | 2 | 12.3 | 10.3 |
|  | Ishite River | M6 | 154 | 132.847 | 33.874 | 0.11 | 1.20 | 0.39 | 2 | 12.2 | 10.3 |
|  | Nagara River | G1 | 230 | 136.949 | 35.656 | 0.27 | 1.40 | 0.42 | 32 | 9.4 | 2.6 |
|  | Hida River | G2 | 720 | 137.288 | 36.018 | 0.27 | 1.70 | 0.42 | 32 | 8.9 | 2.6 |
| Gifu | Kosaka River | G3 | 718 | 137.288 | 35.924 | 0.27 | 1.69 | 0.42 | 32 | 8.9 | 2.6 |
|  | Kurisu River | G4 | 501 | 136.965 | 35.826 | 0.27 | 1.50 | 0.42 | 32 | 9 | 2.6 |
|  | Hirose River | S1 | 261 | 140.626 | 38.320 | 0.24 | 1.20 | 0.30 | 71 | 8.2 | 8.1 |
|  | Natori River | S2 | 315 | 140.593 | 38.270 | 0.24 | 1.20 | 0.30 | 71 | 8.2 | 8.1 |
| Sendai | Natori River | S3 | 850 | 140.864 | 38.797 | 0.24 | 1.40 | 0.30 | 71 | 8 | 8.1 |
|  | Nikkawa River | S4 | 303 | 140.610 | 38.328 | 0.24 | 1.30 | 0.30 | 71 | 8.2 | 8.1 |
|  | Aosita River | S5 | 366 | 140.643 | 38.333 | 0.24 | 1.30 | 0.30 | 71 | 8.2 | 8.1 |
|  | Ohkura River | S6 | 394 | 140.677 | 38.357 | 0.24 | 1.30 | 0.30 | 71 | 8.2 | 8.1 |
|  | Kotonihassamu River | Sa1 | 95 | 141.271 | 43.0557 | 0.12 | 6.00 | 0.73 | 113.6 | 7 | 0.4 |
|  | Nakano River | Sa2 | 200 | 141.254 | 43.069 | 0.12 | 4.00 | 0.73 | 113.6 | 6.7 | 0.4 |
| Sapporo | Ashibetsu River | Sa3 | 305 | 142.135 | 43.316 | 0.12 | 4.00 | 0.73 | 113.6 | 6.7 | 0.4 |
|  | Sorachi River | Sa4 | 188 | 142.271 | 43.473 | 0.12 | 3.00 | 0.73 | 113.6 | 6.9 | 0.4 |
|  | Toyohira River | Sa5 | 247 | 141.254 | 42.956 | 0.12 | 4.00 | 0.73 | 113.6 | 6.7 | 0.4 |
|  | Makomanai River | Sa6 | 116 | 141.355 | 42.988 | 0.12 | 3.00 | 0.73 | 113.6 | 6.9 | 0.4 |
|  | Makomanai River | Sa7 | 298 | 141.321 | 42.908 | 0.12 | 4.00 | 0.73 | 113.6 | 6.7 | 0.4 |

**Additional file 1: Table S2.** Number of individuals per sampling site. Geographical Regions: M, Matsuyama; G, Gifu; S, Sendai; and Sa, Sapporo. Sampling site codes are shown as described in Supplementary Table S1

| sampling site | *Nemoura ovovercia* | *Haploperla japonica* | *Stavsolus japonicus* | *Rhabdiopteryx japonica* | *Obiopteryx femoralis* | *Isoperla nipponica* | *Amphinemoura longispina* |
| --- | --- | --- | --- | --- | --- | --- | --- |
| M1 | 2 | 2 | 2 | 3 | 2 | 2 | 2 |
| M2 |  | 2 | 2 | 2 | 2 | 2 | 2 |
| M3 | 3 | 2 | 3 |  |  | 2 | 2 |
| M4 |  | 2 | 2 |  |  | 2 |  |
| M5 |  |  | 2 |  | 2 |  |  |
| M6 |  |  | 2 |  | 2 |  | 2 |
| G1 | 2 | 2 | 3 | 2 | 2 | 2 | 2 |
| G2 | 3 | 2 | 4 | 3 | 2 | 2 | 2 |
| G3 |  | 2 | 3 |  |  | 2 | 3 |
| G4 |  | 2 | 3 |  | 3 | 2 |  |
| S1 | 2 | 2 | 3 | 2 | 2 | 2 | 2 |
| S2 |  | 2 | 2 | 2 | 2 |  |  |
| S3 | 3 | 2 | 2 | 2 |  | 2 | 2 |
| S4 |  | 2 | 2 |  |  | 2 | 3 |
| S5 |  |  | 2 |  | 2 |  |  |
| S6 |  |  | 2 |  | 2 | 2 |  |
| Sa1 | 2 | 2 | 2 | 2 | 2 | 2 | 2 |
| Sa2 | 3 | 2 | 2 | 2 | 2 | 2 | 2 |
| Sa3 |  |  | 2 | 2 |  | 2 |  |
| Sa4 |  | 2 | 2 |  | 2 |  |  |
| Sa5 |  | 2 |  |  | 2 |  |  |
| Sa6 |  |  | 2 |  |  | 2 | 2 |
| Sa7 |  |  | 3 |  |  |  | 2 |

**Additional file 1**: **Appendix S1.** *de novo* assembly filtering and post-assembly filtering

Using 219 individuals of seven species (*Haploperla japonica*, *Nemoura ovocercia*, *Rhabdiopteryx japonica*, *Obipteryx femoralis*, *Isoperla nipponica*, *Amphinemura longispina*, *Stavsolus japonicus*), we performed a *de novo* assembly using the STACKS v.2.0 pipeline. Different parameter combinations were evaluated. Minimum read depth to create consensus loci (m), number of mistmatches allowed between loci within individuals (M), and number of mismatches allowed between loci within consensus loci (n) were tested (Table S2).

For each parameter combination run, we performed loci data-filtering using the population program in STACKS program (e.i. > 80% occurrence of individuals; allele frequency < 0.05; minimum 30x read coverage; retaining one SNP per locus). We examined the effect of merging alleles from different loci based on two observations, over- and under-merging alleles. A potential cause of over-merging is allowing high number of mismatches within loci and between consensus loci, resulting in an excess of homozygotes. Alternatively, under-merging due too few nucleotide mismatches would result in divert alleles considered as separate loci (Pari *et al*. 2017). In order to assess over- and under-merging of consensus loci, we observed the distribution of observed and expected homozygotes of Hardy Weinberg (HW) equilibrium, which could not be greater than 1:1 expectation; and the F*is* (inbreeding coefficient) distribution, which values greater to zero represent an excess of homozygotes (as proposed by Ravinet *et al*. 2016). Additionally, an excess of homozygotes such as those seeing in F*is* distribution may also cause an incorrect *de novo assembly* by high depth coverage reads (> 30X) (Li, 2014). The Linkage Disequilibrium (LD) test was not applied, as SNPs in LD are not in HW equilibrium observed in the test above.

Our results from different parameter combination showed different number of loci (Table S3). The observed:expected homozygotes and F*is* distribution across loci remained similar across parameter combination runs. The average observed:expected homozygotes was 0.995:0.997 for all the species and all parameter combination. While, the average F*is* ranges 0-0.05 for all the species and all parameter combination. *I*. *nipponica* failed to the construction of loci for the parameter “m10 M3 n2” (example as Table S4), “m10 M3 n4”, and “m10 M3 n8”. The parameters of “m5 M2” and “m10 M3” regarding the number of mismatches allowed between loci (n) resulted in the removal of an average of 11% of the individuals across the species. These results showed that over or under-merged alleles and the influence of coverage reads not affect our analysis output. The F*st* values also were observed, and those showed similar results across species ranged from 0.043-0.1 (example as Table S5). For the main analysis, we opted for the parameter combination m3 M2 n2 for further analysis (Table S6, S7) because of high number of polymorphic loci obtained.

**References**

Li H. Toward better understanding of artifacts in variant calling from high-coverage samples. Bioinformatics. 2014;30: 2843-2851.

Paris, J.R., Stevens, J.R. & Catchen, J.M. Lost in parameter space: a road map for STACKS. Methods Ecol Evol. 2017. doi: 10.1111/2041-210X.12775.

Ravinet, M. *et al*. Shared and nonshared genomic divergence in parallel ecotypes of *Littorina saxatilis* at a local scale. Mol Ecol. 2016;25: 287-305.

**Additional file 1: Table S3.** Number of loci by different parameter combinations evaluated for the *de novo* assembly

| Parameter combination | *Nemoura ovocercia* | *Haploperla japonica* | *Stavsolus japonicus* | *Rhabdiopteryx japonica* | *Obiopteryx femoralis* | *Isoperla nipponica* | *Amphinemoura longispina* |
| --- | --- | --- | --- | --- | --- | --- | --- |
| m3 M2 n2 | 10,715 | 1,871 | 7,273 | 9,856 | 1,4215 | 3,224 | 6,681 |
| m5 M4 n2 | 10,015 | 1,123 | 6,610 | 9,313 | 13,346 | 3,330 | 6,486 |
| m5 M2 n2 | 6,190 | 810 | 4,296 | 4,661 | 7,374 | 2,109 | 3,339 |
| m10 M3 n2 | 4,970 | 917 | 3,245 | 4,539 | 5,905 | 1,619 | 3,069 |
| m3 M2 n4 | 9,995 | 1,440 | 6,434 | 8,265 | 12,145 | 2,917 | 5,073 |
| m5 M4 n4 | 8,690 | 948 | 5,141 | 7,461 | 10,752 | 2,586 | 5,443 |
| m5 M2 n4 | 6,009 | 690 | 3,940 | 4,408 | 7,202 | 1,819 | 3,354 |
| m10 M3 n4 | 4,256 | 826 | 2,831 | 4,160 | 5,028 | 1,450 | 2,929 |
| m3 M2 n8 | 8,721 | 1,261 | 5,931 | 7,292 | 10,413 | 2,731 | 5,001 |
| m5 M4 n8 | 9,500 | 892 | 4,672 | 6,923 | 9,338 | 2,536 | 5,262 |
| m5 M2 n8 | 5,069 | 649 | 3,692 | 3,542 | 5,750 | 1,719 | 2,560 |
| m10 M3 n8 | 3,870 | 680 | 2,677 | 3,439 | 4,267 | 1,312 | 2,522 |

**Additional file 1: Table S4**. Number of loci after final data filtering using the populations program of the STACKS program

|  | m3 M2 n2 | m5 M4 n2 | m5 M2 n2 | m10 M3 n2 |
| --- | --- | --- | --- | --- |
| *Nemoura ovovercia* | 5,073 | 4,151 | 2,938 | 2,478 |
| *Haploperla japonica* | 943 | 490 | 398 | 454 |
| *Stavsolus japonicus* | 3,283 | 3,202 | 1,890 | 1,445 |
| *Rhabdiopteryx japonica* | 4,572 | 3,728 | 2,271 | 2,186 |
| *Obiopteryx femoralis* | 6,445 | 3,523 | 3,022 |  |
| *Isoperla nipponica* | 1,434 | 1,368 | 920 | 721 |
| *Amphinemoura longispina* | 2,568 | 2,320 | 1,050 | 897 |

**Additional file 1:** **Table S5.** F*st* values after final data filtering using the populations program of the STACKS program

|  | m3 M2 n2 | m5 M4 n2 | m5 M2 n2 | m10 M3 n2 |
| --- | --- | --- | --- | --- |
| *Nemoura ovovercia* | 0.106 | 0.11 | 0.15 | 0.11 |
| *Haploperla japonica* | 0.078 | 0.09 | 0.09 | 0.089 |
| *Stavsolus japonicus* | 0.043 | 0.055 | 0.05 | 0.59 |
| *Rhabdiopteryx japonica* | 0.093 | 0.1 | 0.098 | 0.1 |
| *Obiopteryx femoralis* | 0.069 | 0.065 | 0.078 |  |
| *Isoperla nipponica* | 0.081 | 0.087 | 0.088 | 0.087 |
| *Amphinemoura longispina* | 0.120 | 0.13 | 0.12 | 0.132 |

**Additional file 1: Table S6.** Numbers reads per species per region using the parameter combination m3 M2 n2 for the *de novo* assembly after the population program by STACKS

| Species | Matsuyama | Gifu | Sendai | Sapporo |
| --- | --- | --- | --- | --- |
| *Nemoura ovovercia* | 2,064,463 | 4,209,913 | 2,101,856 | 2,016,003 |
| *Haploperla japonica* | 1,114,607 | 3,111,234 | 1,211,838 | 1,045,138 |
| *Stavsolus japonicus* | 1,319,666 | 1,115,217 | 2,111,294 | 2,252,287 |
| *Rhabdiopteryx japonica* | 4,105,786 | 4,010,315 | 4,228,994 | 2,662,303 |
| *Obiopteryx femoralis* | 1,435,539 | 1,577,223 | 2,962,069 | 3,344,019 |
| *Isoperla nipponica* | 2,125,700 | 1,307,239 | 1,329,021 | 3,170,014 |
| *Amphinemoura longispina* | 4,190,319 | 1,093,459 | 260,058 | 3,027,063 |

**Additional file 1: Table S7.** Numbers of loci per species per region using the parameter combination m3 M2 n2 for *de novo* assembly after population program by STACKS

| Species | Matsuyama | Gifu | Sendai | Sapporo |
| --- | --- | --- | --- | --- |
| *Nemoura ovovercia* | 720 | 1,675 | 1,260 | 1,418 |
| *Haploperla japonica* | 227 | 228 | 286 | 202 |
| *Stavsolus japonicus* | 852 | 816 | 811 | 804 |
| *Rhabdiopteryx japonica* | 1,238 | 420 | 1,059 | 1,855 |
| *Obiopteryx femoralis* | 1,037 | 1,437 | 1,804 | 2,167 |
| *Isoperla nipponica* | 355 | 334 | 445 | 300 |
| *Amphinemoura longispina* | 686 | 651 | 550 | 681 |

**
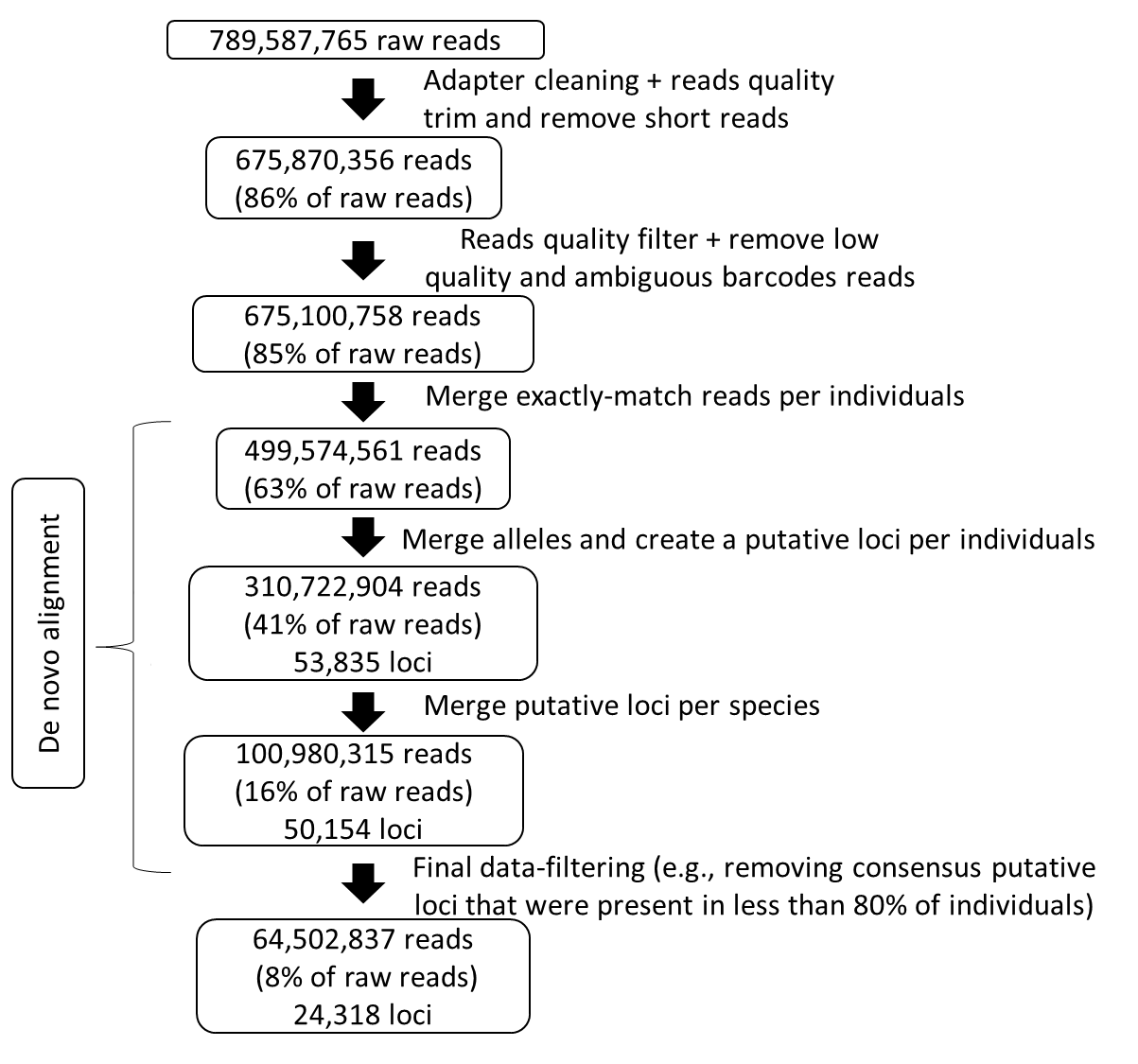
 Additional file 1: Figure S1.** Flow chart of data filtering steps
